# Cushing's Disease is associated with reversible cardiac dysfunction and remodelling assessed by CMR

**DOI:** 10.1186/1532-429X-16-S1-P242

**Published:** 2014-01-16

**Authors:** Roux Charles, Peter Kamenicky, Nadjia Kachenoura, Elie Mousseaux, Philippe Chanson, Alban Redheuil

**Affiliations:** 1 Medical imagery, Paris VI Pierre et Marie Curie, Paris, France; 2 Endocrinology, Université Paris Sud, Krêmelin Bicêtre, France; 3 Medical Imagery, Paris V Descartes, Paris, France

## Background

Cardiovascular complications are the main cause of death in chronic hypercorticism. Cushing's disease (CD) is caused by a benign pituitary gland secreting tumor resulting in endogen hypercorticism. Few ultrasound studies have described cardiac involvement in Cushing's disease, particularly left ventricular (LV) hypertrophy. Hypertrophy has been related to the often associated hypertension and little is known about the effect of high cortisol levels on ventricular mass and function. This is the first comprehensive description of biventricular and left atrial volumes and LV mass assessed by CMR in hypercorticism compared to matched controls. Furthermore, the reversibility of the cardiac phenotype associated with hypercorticism was evaluated after treatment.

## Methods

This is a longitudinal monocentric case control study. All CMR exams were performed between from 2010 to 2013 on 18 consecutive CD patients and 18 asymptomatic controls matched on age, body mass index, and blood pressure range. First CMR was performed upon initial diagnosis and a follow-up exam was done on average 8 months after surgical treatment. Right and left ventricular and atrial volumes, left ventricular mass, thickness, were quantified on SSFP cine CMR and normalized by body surface area. Biventricular and left atrial ejection fractions were calculated. 24-hour urinary free cortisol and glycemia were recorded.

## Results

No patients were lost to follow up. Compared to controls, baseline right and left ejection fractions were significantly altered in CD patients (respectively LV: 65 vs 55%, p < 0.0001; RV: 60 vs. 49%, p < 0.01) mainly due to increased end systolic right and left ventricular volumes in CD patients (LV: 60 vs 39 ml, p < 0.01; RV: 71.5 vs 46 ml, p = 0.0278). Left atrial ejection fraction was significantly lower among CD patients (56 vs 39%, p < 0.01). Average LV wall thickness was markedly increased in basal, mid-ventricular and apical segments (11.2 vs 7.99; 10.3 vs 7.26; 8.45 vs 5.90, p < 0.0001). After treatment, 24-hour urinary free cortisol was normalized (6 vs. 373 μg/24 h, p = 0.0003). LV mass decreased (109 vs 86 g, p = 0.0007), LV and RV stroke volumes increased (LV: 35 vs 42 mL/m, p = 0.0418; RV: 81.9 vs 63.0, p = 0.0294) and left atrial and ventricular function improved (LA: 39 vs 52%, p = 0.0002; LV: 58 vs 55%, p = 0.0294). No death or intercurrent cardiovascular event was observed during the study period.

## Conclusions

CMR allowed to demonstrate infraclinical biventricular cardiac involvement in Cushing's disease with systolic ventricular and atrial dysfunction and concentric LV remodeling that were largely reversible after radical treatment.

## Funding

This study was funded by Université Paris Descartes (V), and by Université Paris Sud.

